# β-Lactam Antibiotics Renaissance

**DOI:** 10.3390/antibiotics3020193

**Published:** 2014-05-09

**Authors:** Wenling Qin, Mauro Panunzio, Stefano Biondi

**Affiliations:** 1 ISOF-CNR Department of Chemistry “G. Ciamician”, Via Selmi, 2 I-40126 Bologna, Italy; E-Mail: wenling.qin2@unibo.it; 2 Allecra Therapeutics SAS, 13, rue de Village-Neuf, F-68300 St-Louis, France

**Keywords:** β-lactam antibiotics, β-lactamase inhibitors, bacterial infections

## Abstract

Since the 1940s β-lactam antibiotics have been used to treat bacterial infections. However, emergence and dissemination of β-lactam resistance has reached the point where many marketed β-lactams no longer are clinically effective. The increasing prevalence of multidrug-resistant bacteria and the progressive withdrawal of pharmaceutical companies from antibiotic research have evoked a strong reaction from health authorities, who have implemented initiatives to encourage the discovery of new antibacterials. Despite this gloomy scenario, several novel β-lactam antibiotics and β-lactamase inhibitors have recently progressed into clinical trials, and many more such compounds are being investigated. Here we seek to provide highlights of recent developments relating to the discovery of novel β-lactam antibiotics and β-lactamase inhibitors.

## 1. Introduction

The emergence and spread of resistance to antibiotics always has accompanied their clinical use. During the past 20 years, however, physicians have experienced a worrisome situation, described by Shlaes [1] and by Pendleton [2], involving a significant increase in morbidity and mortality due to bacterial infections in both community and hospital settings. In particular, two types of strains have compromised the clinical utility of currently available antibiotics and underscored the need for new compounds:

- multidrug-resistant strains (MDR), which are non-susceptible to one or more drugs belonging to ≥3 antimicrobial classes;
- and extremely drug-resistant strains (XDR), which are non-susceptible (or nearly so) to all classes of antimicrobials [3].

The bacteria that constitute the ESKAPE pathogens (***E**nterococcus faecium*, ***S**taphylococcus aureus*, ***K**lebsiella pneumoniae*, ***A**cinetobacter baumanii*, ***P**seudomonas aeruginosa*, and *Enterobacter* spp.) are responsible for about 30%–35% of nosocomial infections, including the vast majority of MDR and XDR strains, leaving physicians with limited therapeutic options [3,4]. In this review we report recent progress in the discovery and development of novel β-lactam antibiotics [5,6] and β-lactamase inhibitors, including β-lactamase inhibitors lacking a β-lactam ring [7,8].

## 2. Discussion

Since 2000 about 20 new antibiotics have been launched, covering five new drug classes for combating bacterial diseases. These five new classes are represented by linezolid (intravenous and oral oxazolidinone, active against Gram-positive cocci, approved 2000), daptomycin (intravenous lipopeptide, active against Gram-positive cocci, approved 2003), retapamulin (topical pleuromutilin, active against Gram-positive cocci, approved 2007), fidaxomicin (oral macrocycle, active against *Clostridium difficile*, approved 2010), and bedaquiline (intravenous diarylquinoline, active against *Mycobacterium tuberculosis*, approved 2012). Table 1 lists new antibiotics approved since the start of the second millennium.

**Table 1:** Antibiotics approved since 2000.

Year approved	Drug name	Chemical structure	*Class*	Bacterial profile	Special features
2000	**Linezolid**	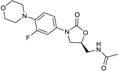	*oxazolidinone*	G+	MRSA
2001	**Telithromycin**	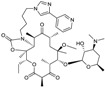	*Macrolide (ketolide)*	G+/some G−	Safety concerns
2002	**Biapenem**	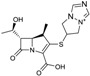	*carbapenem*	G+, G−	Broad spectrum, including many β-lactamase producers
2002	**Ertapenem**	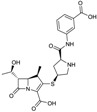	*carbapenem*	G+, G−	Broad spectrum, including many β-lactamase producers
2002	**Prulifloxacin**	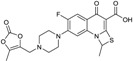	*fluoroquinolone*	G+, G−	Broad spectrum
2002	**Pazufloxacin**	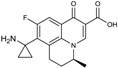	*fluoroquinolone*	G+, G−	Broad spectrum
2002	**Balofloxacin**	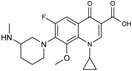	*fluoroquinolone*	G+, G−	Broad spectrum
2003	**Daptomycin**	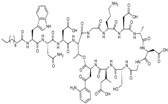	*lipopeptide*	G+	MRSA, VRE
2004	**Gemifloxacin**	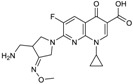	*fluoroquinolone*	G+, G−	Broad spectrum
2005	**Doripenem**	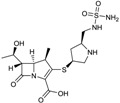	*carbapenem*	G+, G−	Broad spectrum, including many β-lactamase producers
2005	**Tigecycline**	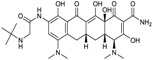	*tetracycline (glycylglycine)*	G+, G−	Broad spectrum
2007	**Retapamulin**	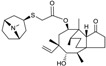	*pleuromutilin*	G+	MRSA
2007	**Garenoxacin**	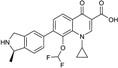	*Quinolone*	G+, G−	Broad spectrum,
2008	**Ceftobiprole medocaril**	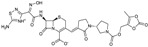	*cephalosporin*	G+, G−	Broad spectrum, MRSA
2008	**Sitafloxacin**	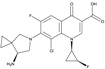	*fluoroquinolone*	G+, G−	Broad spectrum
2009	**Tebipenem pivoxil**	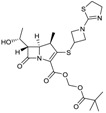	*carbapenem*	G+, G−	Broad spectrum, including many β-lactamase producers
2009	**Telavancin**	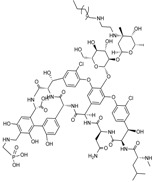	*glycopeptide*	G+	MRSA
2009	**Antofloxacin**	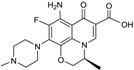	*fluoroquinolone*	G+, G−	Broad spectrum
2009	**Besifloxacin**	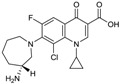	*fluoroquinolone*	G+, G−	Broad spectrum
2010	**Ceftaroline fosaminyl**	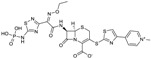	*cephalosporin*	G+, G−	Broad spectrum
2011	**Fidaxomycin**	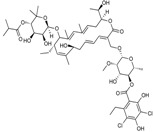	*macrocycle*	G+	*C.difficile*
2012	**Bedaquiline**	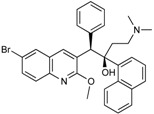	*diarylquinoline*	acid-fast bacteria	*Mycobacterium tuberculosis*

Widespread use of β-lactam antibiotics derives from their efficacy and safety profile, but emergence of new, more aggressive β-lactamases is obliging clinicians to resort to drugs with lower therapeutic windows, such as polymyxins and tigecycline. According to the Pharmaceutical Research and Manufacturers of America, there are 124 products in development for bacterial infections, the vast majority of which are in Phases II and III, with only a few of them being new chemical entities with novel mechanism of action, underlying the difficulties in fuelling the pipeline. To improve the situation and counter bacterial resistance, several government-supported initiatives have been undertaken, including the US Generating Antibiotic Incentives Act (“GAIN”: automatic priority review and an additional 5–7 years of market exclusivity for qualified infectious disease products), and the Innovative Medicines Initiative New Drugs for Bad Bugs (IMI ND4BB), a fund to support clinical development of new antibiotics, particularly those effective against Gram-negative pathogens [9]. Amongst other incentives to have been proposed [10,11,12,13], the Infectious Diseases Society of America launched (2010) its “10 × '20 Initiative” to develop 10 new safe and efficacious systemically administered antibiotics by 2020 [14]. In all of this antibiotic development activity, novel β-lactam antibiotics or β-lactamase inhibitors play a significant role. One avenue of research has aimed at identifying novel β-lactams belonging to well established classes, but with an expanded spectrum, e.g., methicillin-resistant staphylococci (MRSA), *P. aeruginosa*, and/or *A. baumannii*. Another avenue has focused on identifying novel β-lactamase inhibitors that can protect existing β-lactams from emerging β-lactamases, like KPC, OXAs, and metallo-β-lactamases. This approach ought to enable the rejuvenation of old antibiotics that lately have lost their efficacy due to the appearance of new resistance. 

### 2.1. β-Lactam and β-Lactamase Inhibitors in Development

Nearly all recently-developed β-lactam antibiotics have been studied in combination with β-lactamase inhibitors, because bacteria have evolved β-lactamases that hydrolyze (inactivate) all β-lactam classes, including carbapenems. The antibacterial pipeline contains several combinations of novel β-lactamase inhibitors with “old” β-lactam antibiotics and “old” β-lactamase inhibitors with new β-lactam products (Table 2). Presently the most important medical need lies with countering MDR and XDR Gram-negative pathogens [15]. The decline in efficacy of available β-lactams has stimulated the study of creative antibiotic combinations (e.g., carbapenems + polymyxins, mecillinam + amoxicillin/clavulanate) in an effort to identify therapeutic alternatives to monotherapies [16,17,18]; publications on BAL30072, an experimental ESBL-susceptible β-lactam, suggest that for most indications this compound is being positioned as a partner with meropenem (*vide infra*). Due to the limitations of such approaches, that often are unable to overcome the emerging resistance mechanisms, research has focused on other directions, including new β-lactam/β-lactamase inhibitor combinations.

**Table 2:** β-Lactams, β-lactamase inhibitors, and β-lactams/β-lactamase inhibitor combinations in development.

Compounds	*Chemical classes*	Bacteria profile	Indication (Company)	(Pre)-Clinical phase
CXA-201 (ceftolozane/tazobactam)	*cephalosporin/sulfone penam*	G+, G−	cUTI, cIAI; HABP/VABP (Cubist)	III, Completed II
CAZ104 (ceftazidime/avibactam)	*cephalosporin/diazabicyclooctane*	G+, G−	cIAI; UTI (AstraZeneca)	III
CXL (ceftaroline/avibactam)	*cephalosporin/diazabicyclooctane*	G+, G−	MRSA (AstraZeneca)	III
Imipenem/cilastatin/MK-7655	*carbapenem/DHP-I inhibitor/diazabicyclooctane*	G+, G−	UTI and cIAI (Merck)	II
BAL30072	*monobactam*	G+, G−	Gram-negative (Basilea)	I
S-649266 (GSK-2696266)	*cephalosporin*	G+, G−	Gram-negative infections (Shionogi/GSK)	I
ATM-AVI (aztreonam/avibactam)	*monobactam/diazabicyclooctane*	G+, G−	Metallo β-lactamase producers (AstraZeneca)	I
Carbavance (biapenem/RPX7009)	*carbapenem/Boronic acid*	G+, G−	KPC, CRE (The Medicines Company, previously Rempex)	II
TD-1792	*glycopeptide-cephalosporin hybrid*	G+	(Theravance)	II-III
FPI-1465	*diazabicyclooctane*		Fedora	Discovery
Novel β-lactamase inhibitors	*sulphonamides*		(John Hopkins Un.)	Discovery
Novel β-lactamase inhibitors	*Boronic acid*		(John Hopkins Un.)	Discovery
Novel β-lactamase inhibitors	*Boronic acid*		(Therabor Pharmaceuticals)	Discovery
MG96077	*phosphonate-based β-lactamase inhibitor*		(Mirati Therapeutics)	Discovery
CB-027	*cephalosporin*	G+, G−	MRSA, *P. aeruginosa* (Cubist)	Discovery
FSI-1671	*carbapenem*	G−	(FOB Synthesis Inc.)	Discovery

#### 2.1.1. CXA-201

CXA-101 (ceftolozane) is a cephalosporin [19] discovered in 2004 by the Fujisawa Pharmaceutical Co., Ltd. (now Astellas, Chertsey, UK) and originally named FR264205 [20,21]. The compound is particularly active against MDR *P. aeruginosa*, including isolates from chronically-infected cystic fibrosis patients [22], due to enhanced affinity of the β-lactam for the PBPs of this species, reported stability to many β-lactamases including AmpCs (but not ESBLs, KPC carbapenemases, or metallo-β-lactamases), and indifference to efflux pumps [23,24,25,26]. However, it also has (relatively) poor activity towards other Gram-negative pathogens, as well as towards multidrug-resistant Gram-positive cocci and anaerobes. Ceftolozane MICs against clinical isolates of *P. aeruginosa* are 8- to 16-fold lower than those of ceftazidime [23,24], and are little affected by MexAB-OprM overexpression and/or OprD deletion. Susceptibility of ceftolozane to ESBLs as evidenced by the 4- to 128-fold increase in MIC by producers of this β-lactamase [27].

**Figure 1:**
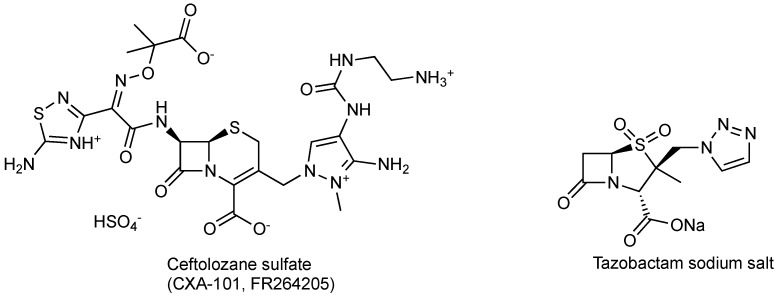
Chemical structure of CXA-101 and tazobactam.

CXA-101 (Figure 1) is a 2:1 (*w*/*w*) combination of ceftolozane and tazobactam, an old (ca. 1990) sulfone penam β-lactamase inhibitor, that is being developed by Cubist. CXA-101 is in Phase 3 trials for treatment of complicated urinary tract infections (levofloxacin as comparator), and for treatment of complicated intra-abdominal infections (meropenem as comparator). Addition of tazobactam at a fixed concentration of 8 µg/mL restored the *in vitro* susceptibility of 93% of ESBL producers and 95% of the AmpC overproducers examined. However, tazobactam was unable to lower MICs, for Enterobacteriaceae producing KPCs, [28] and CXA-101 does not show better activity than ceftolozane alone against *P. aeruginosa*. Cubist recently filed a patent covering use of ceftolozane/tazobactam (2:1) for treating pulmonary infections [29].

#### 2.1.2. CAZ104

**Figure 2:**
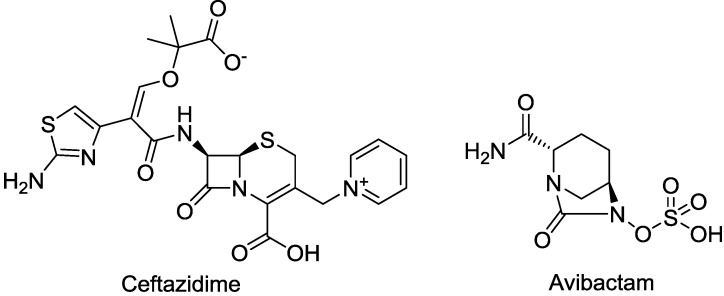
Chemical structure of **CAZ104** and avibactam.

Ceftazidime is a third-generation cephalosporin with generally good activity against Gram-negative pathogens, including *P. aeruginosa* and *Enterobacteriaceae* [30]. Ceftazidime currently is being developed in combination with avibactam, a diazabicyclooctane (a non-β-lactam β-lactamase inhibitor) which inhibits preferentially class A β-lactamases, including ESBLs and KPCs [31], with lesser but still clinically useful activity against AmpCs; the compound has variable activity towards OXAs [32], but is inactive towards metallo-β-lactamases. Figure 2 shows the chemical structures of ceftazidime and avibactam, the constituents of CAZ104 [33].

**Figure 3:**
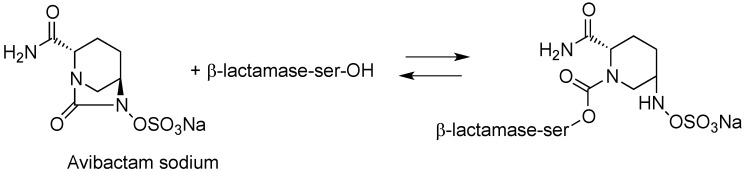
Mechanism of action of avibactam.

Avibactam forms a covalent bond with β-lactamases that is slowly reversible, regenerating the avibactam molecule and catalytically active β-lactamase enzyme (Figure 3) [34,35,36].

Avibactam reduces the MICs of ceftazidime (2- to 16-fold) towards AmpC-derepressed mutants of *P. aeruginosa*, and against *P. aeruginosa* mutants producing the PER-1. It is not effective against *P. aeruginosa* strains producing OXA ESBLs or VEB-1 [31,37,38,39]. Avibactam does not reduce MICs of ceftazidime towards carbapenem-resistant *A. baumannii*. AstraZeneca has patents covering a process for preparing DBOs (including avibactam) [40], a novel crystalline forms of avibactam [41], and use of DBOs as diagnostic agents [42]. Additional patents cover the preparation of chiral avibactam and useful chiral intermediates [43].

#### 2.1.3. CPT (Ceftaroline)

**Figure 4:**
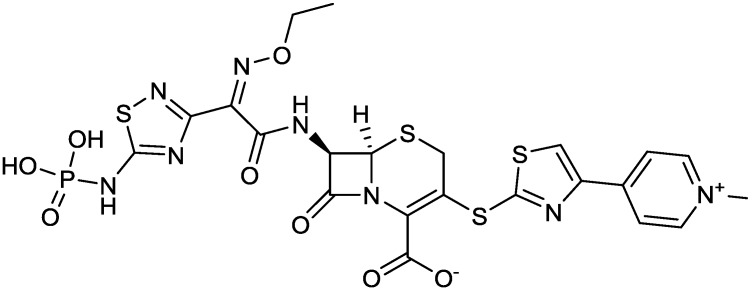
Chemical structure of ceftaroline fosamil.

Ceftaroline fosamil (Figure 4), is a cephalosporin prodrug whose active principle, ceftatoline (CPT), is active against MRS and drug-resistant *S. pneumoniae* [44]. This compound has the distinction of being the first anti-MRS β-lactam to be marketed in the USA (2010), where it received FDA approval for treatment of acute bacterial skin and skin structure infections (SSSI) and for community-acquired pneumonia (CAP) [45]. Ceftaroline shows good clinical efficacy [46] against methicillin-resistant *Staphylococcus aureus* (MRSA) due to its ability to bind to PBP2A.

Avibactam demonstrated synergy with ceftaroline against some β-lactamase-producing anaerobes (*Bacteroides fragilis*, *Prevotella* spp., and *Finegoldia magna* cultured from diabetic foot infections [47]). The antistaphylococcal activity of ceftaroline makes it a good partner antibiotic for certain polymicrobial wound infections where MRSA may be a dominant pathogen [48]. The combination ceftaroline fosamil/avibactam currently is in Phase III trials [49,50].

#### 2.1.4. Imipenem/Cilastatin/MK-7655

**Figure 5:**
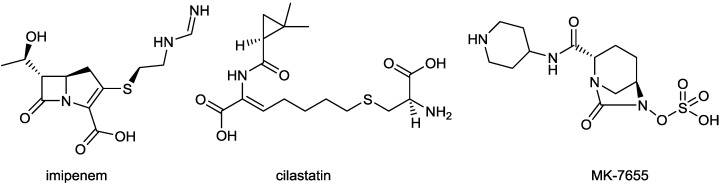
Chemical structures of imipenem, cilastatin, and MK-7655.

Imipenem, the first carbapenem to reach the market (invented and developed by Merck), is a potent, broad-spectrum β-lactam with antipseudomonas activity. It lacks a β-methyl substitutent at position 1 and, accordingly, is not stable to human renal dehydropeptidase I; therefore, imipenem is marketed in combination with cilastatin, a dehydropeptidase inhibitor specifically developed by Merck as a companion to imipenem. MK-7655 is a DBO β-lactamase inhibitor [51] that, combined with imipenem [52], showed good activity against imipenem-resistant Gram-negative isolates *in vitro* [53]. The triple combination of imipenem/cilastin/MK-7655 (Figure 5) poses challenges in terms of a balanced pharmacokinetics, but provides improved activity against some carbapenemase-resistant *P. aeruginosa* and Enterobacteriaceae [54].

**Figure 6:**
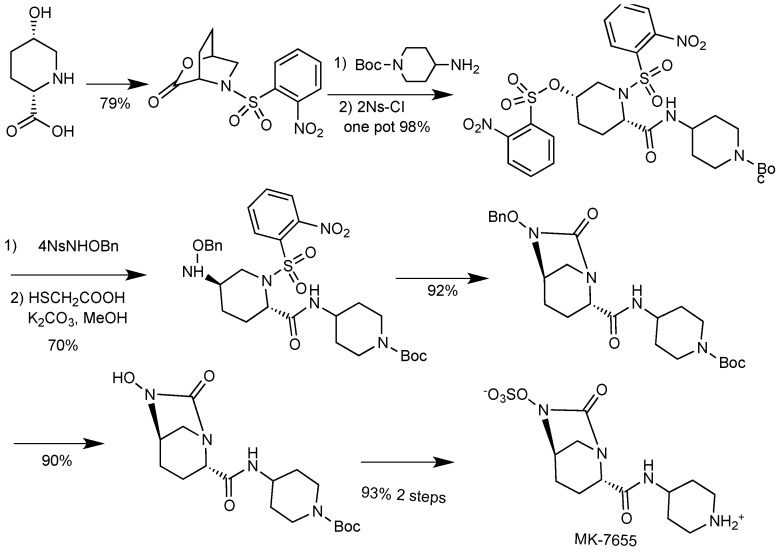
Reaction scheme for the improved synthesis of MK-7655.

A short, scalable, cost-effective route for production of MK-7655 recently has been reported (Figure 6) [55,56]. Synthesis was made possible through the availability of optically pure cis-5-hydroxypipecolic acid, obtained by enzymatic oxidation of pipecolic acid [57]. This route provides pure, crystalline MK7655 in 8 steps, with a 42% overall yield, allowing for a more cost-effective manufacture of the DBO on a multi-kilogram scale.

#### 2.1.5. BAL30072

**Figure 7:**
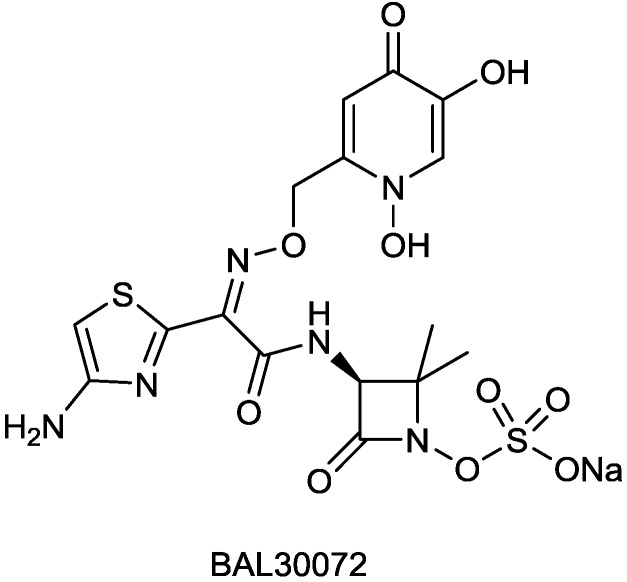
Chemical structures of BAL30072.

BAL30072 (Figure 7) is a monobactam developed by Basilea Pharmaceutica AG and currently in Phase I trials [58]; it is a slight structural modification of tigemonam, where the acetate group has been replaced by dihydroxypyridone, the siderophoric moiety earlier used in the experimental monobactam PTX2416 [59]. BAL30072 is taken up by bacteria *via* one of their iron transport systems [60]; like other monobactams (e.g., aztreonam), BAL30072 is stable towards metallo-β-lactamases and has some inhibitory activity towards class C β-lactamases, though the latter is unlikely to translate into clinical efficacy against most AmpC-producing pathogens. BAL30072 was active against multidrug-resistant (*P. aeruginosa*, *Acinetobacter* spp., *Burkholderia* spp. and *Stenotrophomonas maltophilia*). It was active against 70% of the carbapenem-resistant *Enterobacteriaceae* strains tested [61]. In combination with different carbapenems, BAL30072 showed synergism against several Enterobacteriaceae and *P. aeruginosa* [62]. In 2013 Basilea received a contract potentially totaling US$17 million from the United States Biomedical Advanced Research and Development Authority (BARDA) to continue Phase 1 studies of the safety and tolerability of BAL30072, alone and in combination with carbapenems. It is likely that its further development will involve its combination with carbapenems to extend their activity towards metallo β-lactamases producers [63].

#### 2.1.6. S-649266 (GSK-2696266)

GlaxoSmithKline is developing a cephalosporin, S-649266 (GSK2696266), with Shionogi. The compound is reportedly active against Gram-negative bacteria, including New Delhi metallo-beta-lactamase-1 (NDM-1) producers [64].

#### 2.1.7. ATM-AVI

**Figure 8:**
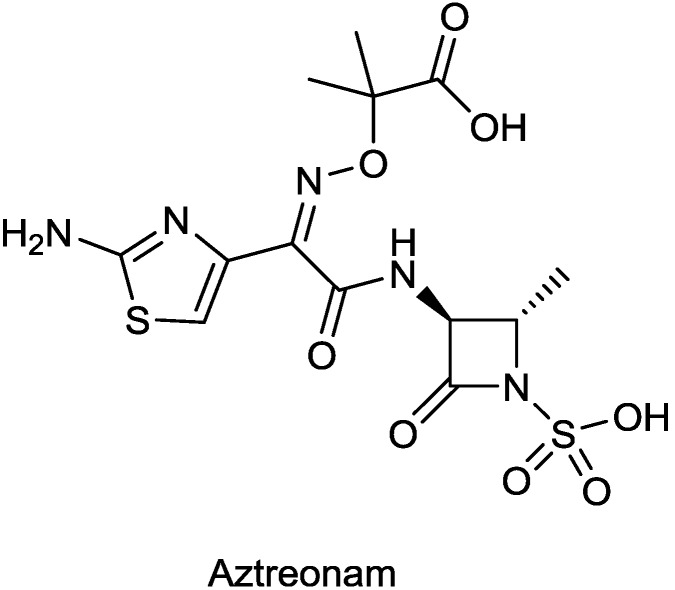
Chemical structure of aztreonam.

ATM-AVI is a combination [65] of aztreonam (Figure 8), a monobactam launched in the US in 1984, and the DBO β-lactamase inhibitor avibactam, under development by AstraZeneca. ATM-AVI was evaluated in a Phase I trial (NCT01689207) but the study was recently suspended because of poor participant recruitment [9].

#### 2.1.8. Biapenem/RPX7009

**Figure 9:**
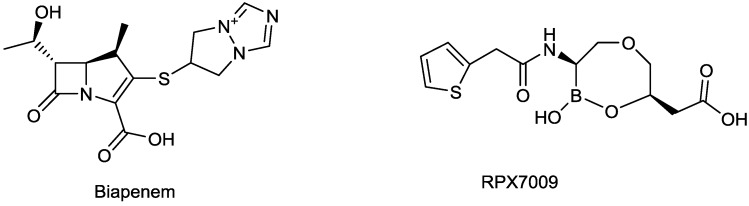
Chemical structures of Carbavance.

Biapenem is a broad-spectrum carbapenem, marketed principally in Japan since 2002, with good activity against *S. pneumoniae*, methicillin-susceptible *Staphylococcus aureus*, *A. baumannii*, ESBL-producing Enterobacteriaceae and, albeit to a lesser extent, *P. aeruginosa* [18,66]. Rempex, recently acquired by The Medicines Co., developed Carbavance (Figure 9), a combination of biapenem with a boronic acid β-lactamase inhibitor, RPX7009 [67]. Carbavance reportedly is active against β-lactamase-producing Gram-negative bacteria, including KPC producers [68,69].

#### 2.1.9. TD-1792

**Figure 10:**
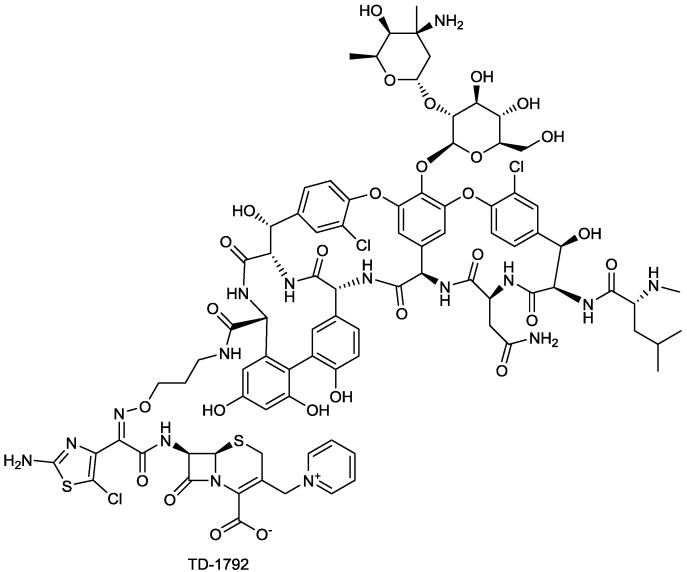
Chemical structure of **TD-1792**.

The antibacterial activity of TD-1792 (Figure 10), a hybrid glycopeptide-cephalosporin antibiotic invented by Theravance, is restricted to Gram-positive pathogens, and has completed a Phase II cSSSI trial [4]. TD-1792 is highly potent towards MRSA and vancomycin-intermediate S*taphylococcus aureus* (VISA). TD-1792 was designed to allow the molecule to interact and inhibit simultaneously two molecular targets involved in murein biosynthesis that are in close proximity to one another, with the expectation that such a strategy would raise significantly the statistical barrier to resistance development [70].

#### 2.1.10. FPI-1465

**Figure 11:**
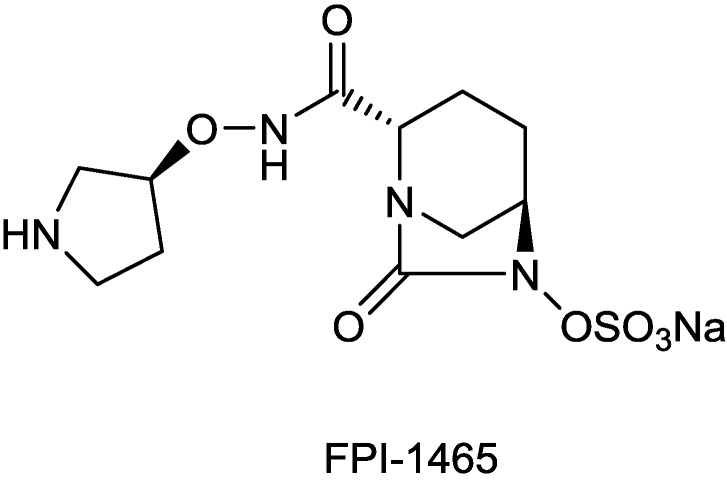
Chemical structure of FPI-1465.

FPI-1465 (Figure 11) is a DBO β-lactamase inhibitor, invented by Fedora Pharmaceuticals, which differs from avibactam only by the presence of a (3S)-pyrrolidin-3-yl oxycarbamate moiety. The compound presently is undergoing preclinical testing [71]. FPI-1465 is inhibitory towards ESBL enzymes produced by Enterobacteriaceae species, such as CTX-Ms, as well as towards plasmid-encoded AmpCs, and it showed synergistic effects when combined with aztreonam and with ceftazidime [71,72,73].

#### 2.1.11. Sulfonamido Boronic Acids

**Figure 12:**
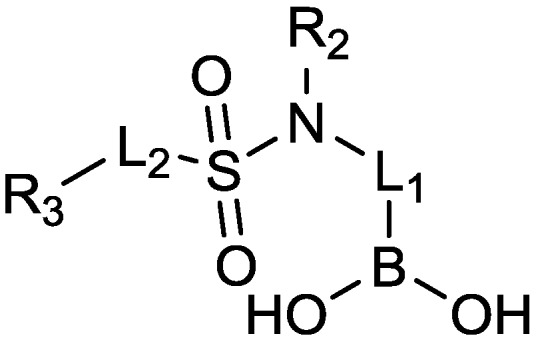
General structure of compounds claimed in WO2013/056079.

The Regents of the University of California and the Università degli studi di Modena e Reggio Emilia have filed a patent, WO 2013/056163, covering novel sulfonamido boronic acids (Figure 12) with β-lactamase inhibitory activity, particularly towards CTX-Ms and AmpCs. [74] One of these compounds, “CR161”, when combined with cefotaxime reduced *E. coli* MICs from 8–128 µg/mL to 0.5–1 µg/mL and also proved active in a mouse model of bacteremia. When mice were infected with a clinical isolate of *E. coli* overproducing AmpC (cefotaxime MIC, 32 µg/mL), then treated i.p. with relatively high doses of either cefotaxime (50 mg/kg) or cefotaxime/CR161 (50/200 mg/kg 0.5, 3.5, and 6.5 h post-infection, survival in response to cefotaxime was 15%, whereas survival in response to cefotaxime/CR161 was 65% by 5 days. Five-days survival following administration of imipenem (50 mg/kg) was 90% [75].

**Figure 13:**
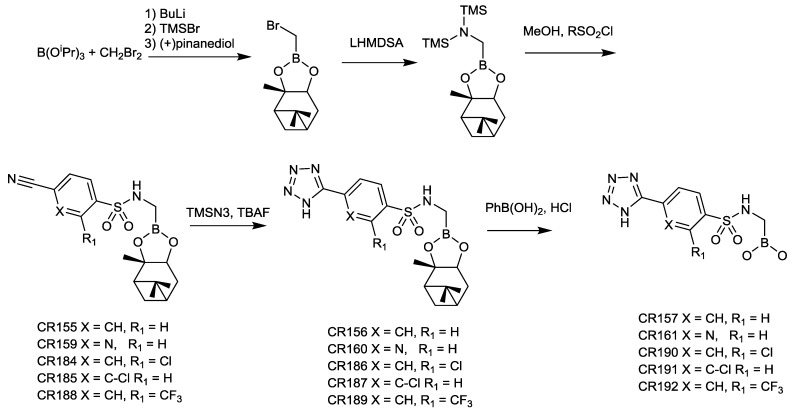
Synthesis of some sulfonamido boronic acids.

Figure 13 shows the reported synthesis of CR161 and some closely related analogues. Starting from triisopropylboronate, bromomethylation using dibromomethane and buthyl lithium, followed by transesterification with (+)-pinanediol, gave (+)-pinanediol bromomethaneboronate. This intermediate was treated with lithium bis(trimethylsilyl)amide to provide the corresponding (+)-pinanediol *N*-bis(trimethylsilyl)aminomethaneboronate. Sulfonylation using the corresponding sulfonyl chlorides in dry methanol gave cyano intermediates CR155, CR159, CR184, CR185, and CR188, which were further reacted with trimethylsilyl azide to yield the corresponding tetrazoles. Finally, transesterification with phenylboronic acid produced the corresponding sulfonylamido boronic acids.

#### 2.1.12. Arylboronic Acids

**Figure 14:**
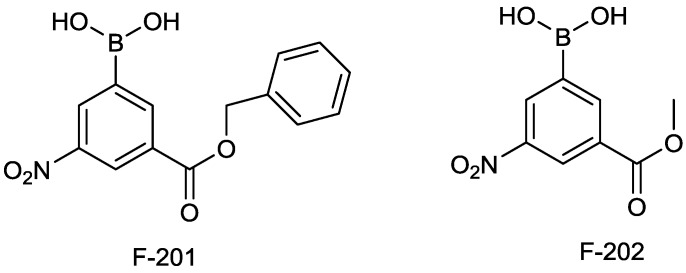
Chemical structure of arylboronic acids.

The John Hopkins University is offering to out-licence a series of arylboronic acids (Figure 14), originally patented by Fulcrum Pharmaceuticals in 2004, showing activity against TEM-1 and AmpC β-lactamases (US7,183,267, also filed as EP1635812).

#### 2.1.13. Triazole-Substituted Boronic Acids

**Figure 15:**
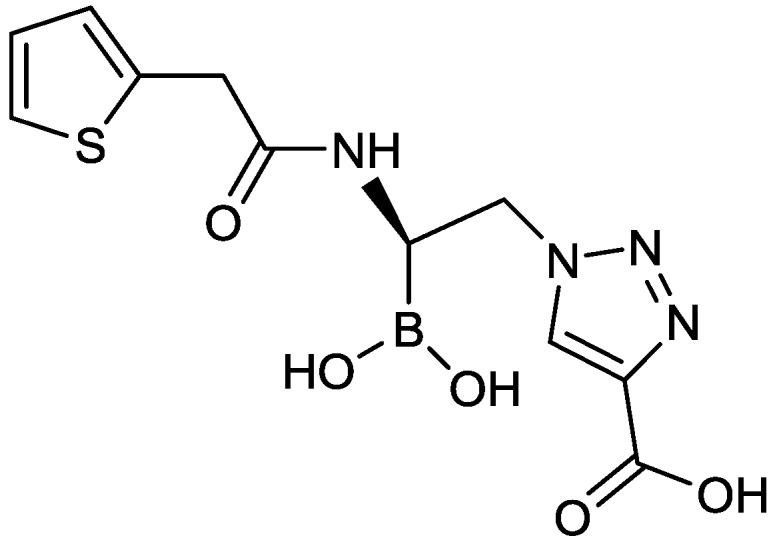
Chemical structure of arylboronic acids.

Therabor Pharmaceuticals patented a series of triazole-substituted boronic acids (Figure 15), some of which inhibited class A and class C β-lactamases, including KPC-2, and displayed synergism with ampicillin towards several strains of β-lactamase producing *E. coli* [76].

#### 2.1.14. Diazabicyclooctane Nitrile

**Figure 16:**
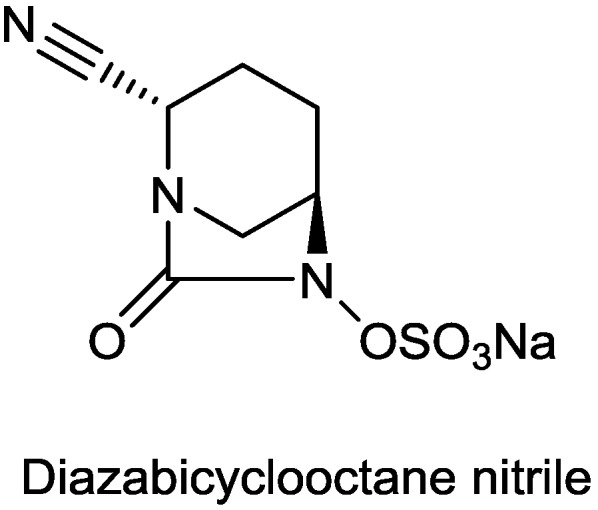
Chemical structure of diazabicyclooctane nitrile.

Wockhardt, Ltd. reported a DBO nitrile (Figure 16) with inhibitory activity comparable to avibactam, and showing synergism with meropenem against oxacillinase-producing strains of *A. baumannii* [77].

**Figure 17:**
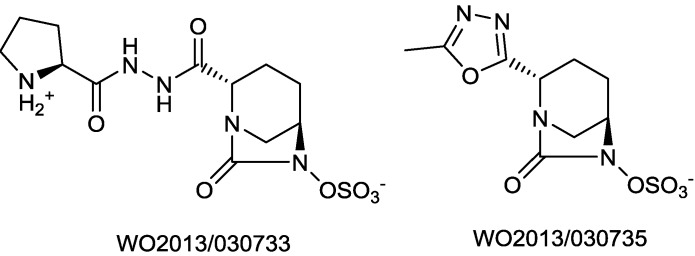
Chemical structure of antibacterial diazabicyclooctanes.

Other novel diazabicycooctanes, with intrinsic activity against *P. aeruginosa* and/or *E. coli*, have been described by Wockhardt (Figure 17) [78,79].

#### 2.1.15. Sulfonamido β-lactamase Inhibitors: John Hopkins University

**Figure 18:**
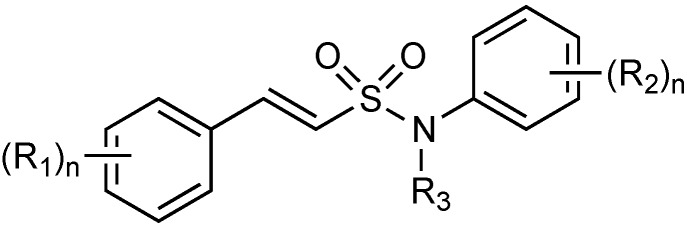
General structure of sulfonamido β-lactamase inhibitors.

Johns Hopkins University has filed patent on a series of sulfonamide β-lactamase inhibitors showing activity against class A, C and B enzymes (Figure 18) [80].

**Figure 19:**
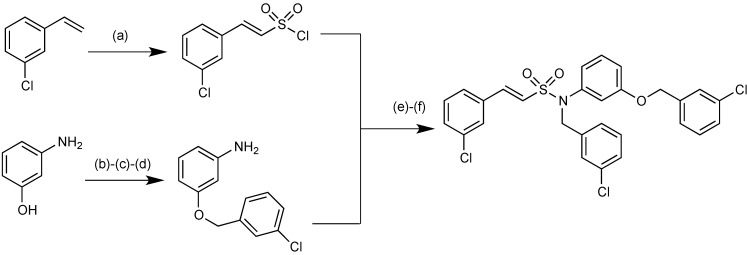
Chemical synthesis of representative sulphonamido β-lactamase inhibitors.

The synthesis of representative sulfonamido β-lactamase inhibitors is shown in Figure 19. The initial step is synthesis of (*E*)-2-(3-chlorophenyl)ethene-1-sulfonyl chloride from 3-chlorostyrene and sulfuryl chloride. In parallel, 3-aminophenol was protected at the nitrogen using Boc anhydride and *O*-alkylated using 3-chlorobenzylbromide. Deprotection of the nitrogen, using trifluoroacetic acid, afforded the *O*-alkylated aniline in 83% yield. The aniline and sulfonyl chloride were then coupled to produce (*E*)-*N*-(3-((3-chlorobenzyl)oxy)phenyl)-2-(3-chlorophenyl)ethene-1-sulfonamide, which was *N*-alkylated using sodium hydride and 3-chlorobenzylbromide to afford the final product in 11% yield.

*Reagents and conditions*: (a) SO_2_Cl_2_, DMF, 55 °C, 5 h; (b) (Boc)_2_O, THF, 61 °C, 24 h; (c) K_2_CO_3_, Nal, DMF, m-chlorobenzyl bromide, 79 °C, 28 h; (d) TFA, CH_2_Cl_2_, RT, 0.5 h; (e) 1 M Na_2_CO_3_, THF/H_2_O, RT, 3.5 h; (f) NaH, m-chlorobenzyl bromide, THF, RT, 11 h.

#### 2.1.16. MG96077

**Figure 20:**
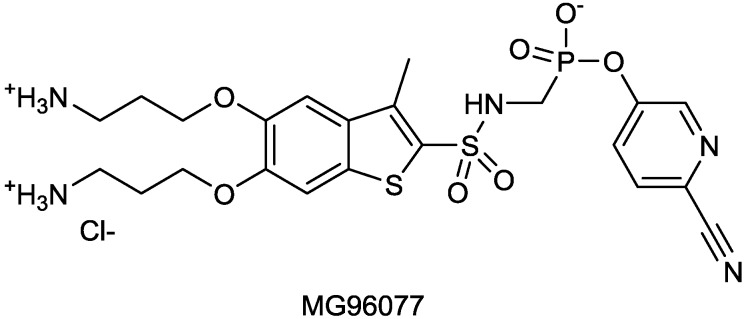
Chemical structure of phosponate β-lactamase inhibitors.

Mirati Therapeutics has identified MG96077, a non-β-lactam phosphonate-based β-lactamase inhibitor, inhibitory towards class A and class C β-lactamases (Figure 20).

#### 2.1.17. CB-027

**Figure 21:**
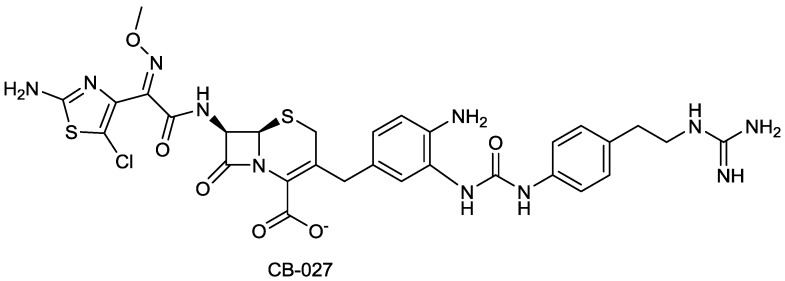
Chemical structure of CB-027.

CB-027 (Figure 21) is reported by Cubist to be an ultra-broad spectrum cephalosporin with *in vitro* activity against both MRSA and *P. aeruginosa*. The compound is claimed to be have *in vivo* activity against clinical isolates of MRSA comparable to that of vancomycin and of ceftaroline, and against ceftazidime-resistant *P. aeruginosa* and *K. pneumonia* [81].

#### 2.1.18. FSI-1671

**Figure 22:**
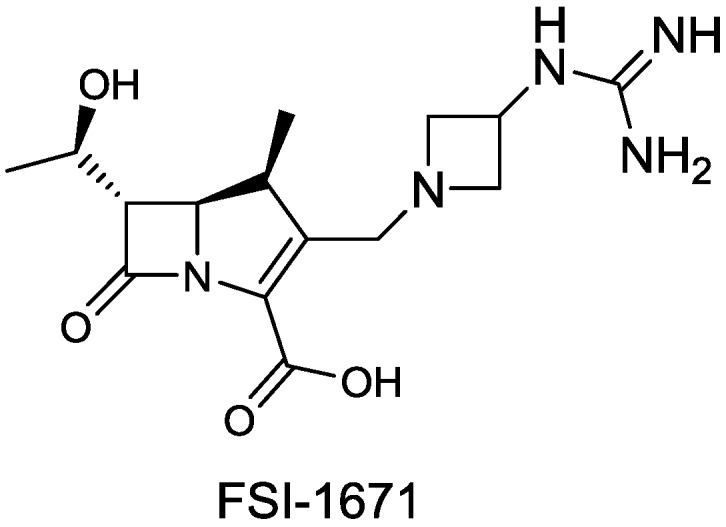
Chemical structure of FSI-1671.

FSI-1671 (Figure 22) is a novel carbapenem, invented by FOB Synthesis, with improved *in vitro* activity against *A. baumannii*, including MDR strains. Joo *et al.* have observed synergism towards MDR strains of *A. baumannii* for FSI-1671 in combination with sulbactam, a β-lactamase inhibitor with intrinsic activity against *Acinetobacter* spp. [82].

#### 2.1.19. Antibacterial Diazabicyclooctanes

Novel diazabicycooctanes having intrinsic antibacterial activity against *P. aeruginosa* and/or *E. coli* have been patented by Wockhardt (Figure 17) [80,81,83].

## 3. Conclusions

The interest for novel β-lactam antibiotics or β-lactamase inhibitors has recently boosted with several companies actively involved in the development of new molecules. The antibacterial pipeline contains several combinations of novel β-lactamase inhibitors with “old” β-lactam antibiotics and “old” β-lactamase inhibitors with new β-lactam products. The main goal is to achieve antibacterial efficacy against multidrug resistant pathogens. In particular, two new families of β-lactamase inhibitors devoid of β-lactam structure are emerging, namely Diazabicycooctanes (DBOs) and boronic acids (see Table 2). These novel β-lactamase inhibitors have been combined with cephalosporins and carbapenems and have shown activity against β-lactamase-producing Gram-negative bacteria, including KPC producers. Despite the progress made so far, activity against certain class A (ESBL, KPC), class D (OXA) and class B (NDM) β-lactamases remains a challenge. Overall β-lactam antibiotics will continue to play an important role in the treatment of difficult infections, particularly addressing multidrug resistant pathogens. It is important to note that after more than 70 years of clinical use, β-lactam antibiotics are still widely prescribed because of their efficacy and safety profile.

Medicinal chemistry research in this field is intense, particularly in the β-lactamase inhibitors and more molecules are expected to enter in the pipeline.
